# T cell Repertoire Profiling and the Mechanism by which HLA-B27 Causes Ankylosing Spondylitis

**DOI:** 10.1007/s11926-022-01090-6

**Published:** 2022-10-05

**Authors:** Jose Garrido-Mesa, Matthew A. Brown

**Affiliations:** 1 Department of Medical and Molecular Genetics, Faculty of Life Sciences and Medicine, King’s College London, London, England; 2 Genomics England, Charterhouse Square, London, EC1M 6BQ England

## Abstract

**Purpose of Review:**

Ankylosing spondylitis (AS) is strongly associated with the HLA-B27 gene. The canonical function of HLA-B27 is to present antigenic peptides to CD8 lymphocytes, leading to adaptive immune responses. The ‘arthritogenic peptide’ theory as to the mechanism by which HLA-B27 induces ankylosing spondylitis proposes that HLA-B27 presents peptides derived from exogenous sources such as bacteria to CD8 lymphocytes, which subsequently cross-react with antigens at the site of inflammation of the disease, causing inflammation. This review describes findings of studies in AS involving profiling of T cell expansions and discusses future research opportunities based on these findings.

**Recent Findings:**

Consistent with this theory, there is an expanding body of data showing that expansion of a restricted pool of CD8 lymphocytes is found in most AS patients yet only in a small proportion of healthy HLA-B27 carriers.

**Summary:**

These exciting findings strongly support the theory that AS is driven by presentation of antigenic peptides to the adaptive immune system by HLA-B27. They point to new potential approaches to identify the exogenous and endogenous antigens involved and to potential therapies for the disease.

## Introduction

Adaptive immunity is orchestrated by peptides (epitopes or antigens) presented by histocompatibility antigens (HLAs) on antigen-presenting cells (APCs). This peptide-HLA (pHLA) pair is specifically recognised by immune receptors of lymphocytes (T cell — TCR; B cells — BCR or immunoglobulins). The sum of all immune receptors of one individual is termed the immune repertoire, which provides coverage against the diverse epitome. The epitome and immune repertoire define the host and the potential symbionts in immunological terms and changes greatly with the onset and progression of diseases. This is the reason why studying the immune repertoire is gaining more and more interest within immune disorders/IMIDs. By providing a global picture of the adaptive immune system, repertoire analyses have potential in both fields, as diagnostic biomarkers and for therapeutic development.

Ankylosing spondylitis (AS) is one of such pathologies where immune repertoire studies could make a significant impact. AS is the prototypic disease of a group of related disorders termed ‘seronegative spondyloarthropathies’ that also includes psoriatic arthritis, reactive arthritis (in response to bacterial urinary or gastrointestinal infection) and arthritis complicating inflammatory bowel disease. In total, these disease affects ~ 2–3% of European-descent populations and at least 1% of Asian populations [1]. AS causes significant back pain, stiffness, reduced function and eventual fusion of the spine and pelvis. AS first presents in early adulthood, initially with no changes visible on plain radiography of the sacroiliac joints (termed ‘non-radiographic axial spondyloarthritis’ (nr-axSpA), with characteristic sacroiliac radiographic changes developing subsequently and defining development of AS itself. Common extraskeletal associations of AS include acute anterior uveitis, inflammatory bowel disease and psoriasis. AS has a lifelong detrimental impact on patients, with depression and anxiety correlating with disease activity measures [2], absenteeism from work and high unemployment rates (40%). Furthermore, AS is also associated with early mortality [3], and current treatments do not change long-term prognosis.

AS is highly familial, with an increased risk in siblings of AS patients 82 × higher than the disease prevalence in the general community [4]. Twin studies [5, 6] suggest that > 90% of AS susceptibility is genetic in origin whilst the environmental trigger is likely ubiquitous. To date, 116 independent genomic loci having been robustly identified, contributing ~ 30% of the overall genetic risk, with ~ 20% being due to HLA-B*27 [7, 8, 9, 10, 11, 12•], one of the strongest genetic associations seen with any polygenic human disease (OR = 60, *P* < 10^−300^) (observed in > 80% of cases [13]). GWAS findings show that AS involves both the adaptive and innate immune systems, and that the IL-23 and TNF pathways are the major effector pathways involved [14].

**Fig. 1:**
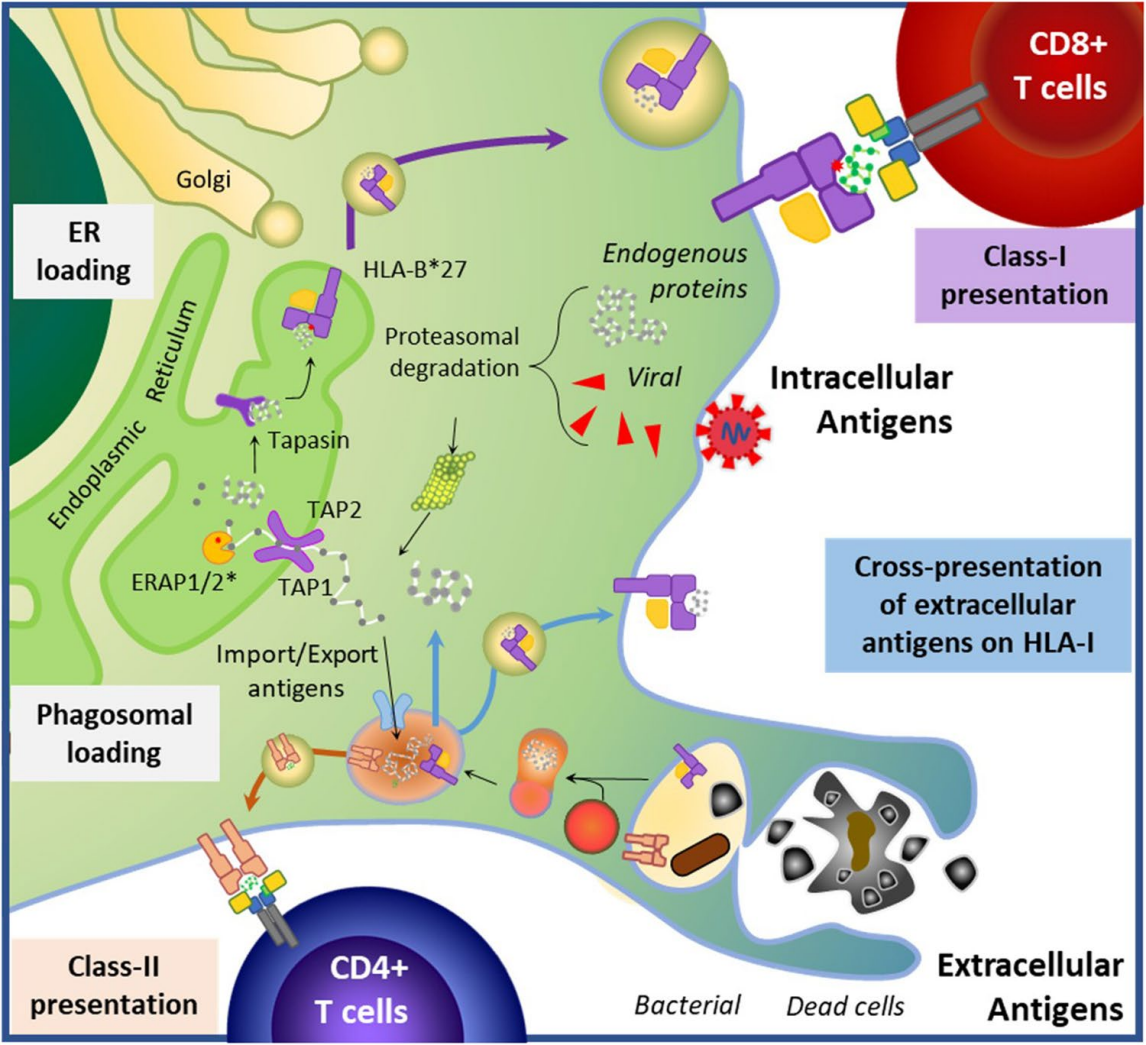
Antigen presentation pathways altered by HLA-B*27 and ERAP1/2 epistasis: Generally, intracellular or extracellular antigens are presented on lymphocytes by HLA Class-I or Class-II molecules, respectively. Antigen-presenting cells can also mobilize extracellular antigens (such as those from phagocytosed bacteria or dead cells) for class-I presentation (termed cross-presentation) and generation of cytotoxic responses. Upon phagosomal or proteasomal degradation, antigens can be directly loaded or imported to the endoplasmic reticulum (ER) by TAP1/TAP2 proteins. Aminopeptidases at the ER (ERAP1/2) trim imported proteins, which are then loaded into the HLA molecule for presentation, aided by Tapasin. The peptide-HLA complex is then exported to the surface for recognition by its cognate TCR. The interaction between altered HLA-B*27 and ERAP1/2 leading to AS (epistasis) was the first example of gene–gene interaction identified in any human disease, and results in an altered peptidome presented to cytotoxic T lymphocytes, consistent with the arthritogenic peptide hypothesise

HLA-B27 is a member of the HLA Class I family of MHC genes whose role is to present peptide antigens to CD8 T cells (Fig. 1). In AS, psoriasis and Behcet’s disease, there is robust evidence of gene–gene interaction between the HLA-I risk allele and *ERAP1*, indicating that they must operate closely together to influence disease risk [8, 15, 16]. The M1-aminopeptidase genes *ERAP1* and/or *ERAP2* are involved in the HLA-I antigenic processing and presentation (APP) pathway. These *ERAP1* variants in AS patients exhibit significantly increased catalytic activity, resulting in over-trimming and creation of ‘unusual peptides’ presented by HLA-B27, which may not normally be generated in healthy situations. AS-associated ERAP2 variants break down peptides of 8 amino acids, and ERAP1 variant peptides longer than 9 amino acids [17]. This indicates that the peptides bound by HLA-B27 involved in AS-aetiopathogenesis are 9 amino acids in length, even though HLA-B27 has been shown to be able to present longer peptides.

**Fig. 2:**
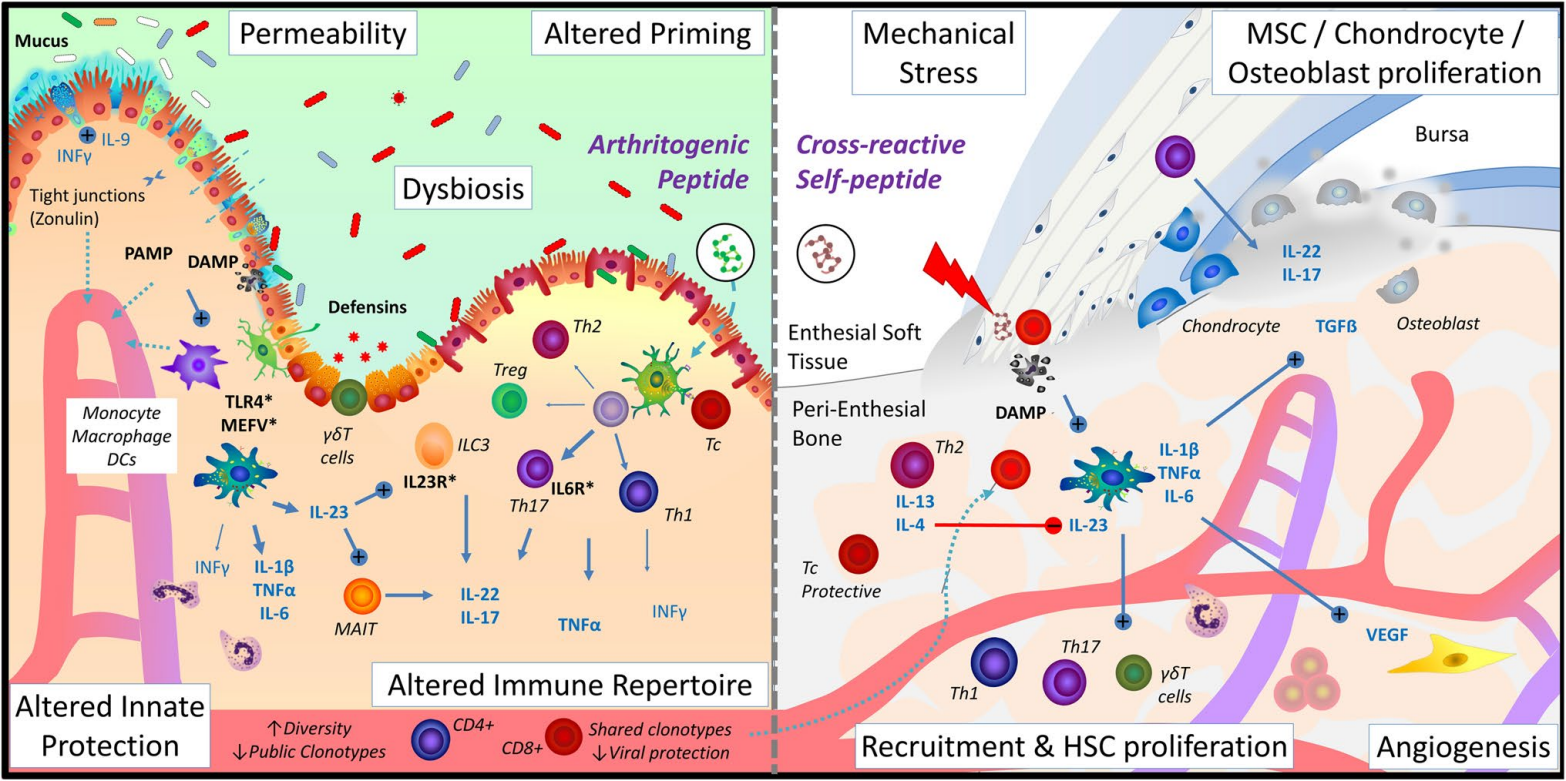
Immune regulation of the joint-gut-microbiome axis in AS. Left: Intestinal inflammation is a frequent comorbidity of AS, and GWAS studies have identified genetic susceptibility among genes regulating important pathways for mucosal immune homeostasis (highlighted with * in the figure). A reduction in the physicochemical properties of the mucosal barrier (intercellular tight junctions, mucus layer and antimicrobial compounds, such as defensins) leads to increased permeability (leaky gut) and immune activation by pathogen (PAMP) and danger-associated molecular patterns (DAMP). Genetic variants within key immune regulatory genes (*TLR4*, *MEFV*, *IL23R*, *IL6R*, *IL7R)* result in an altered immune response, where immune cells show both deficient reactivity and persistent activation. Together with alterations in antigen-presentation pathways (Fig. 1), this immune imbalance leads to altered immune priming and circulating repertoire, which likely result in the reduction of protective public clonotypes (i.e. reduced CMV and EBV-specific expansions) and the generation of disease-associated clonotypes. Right: Disease-associated clonotypes generated against “unusual” antigens presented on this condition can recognise self-antigens due to TCR degeneracy and peptide mimicry, thus initiating a cytotoxic response at the tissue location where the self-antigen is found (the enthesis). The enthesial tissue, also impacted by mechanical stress, becomes inflamed, initiating a pathologically deleterious process leading to stromal cell proliferation and calcification, as observed in the latter stages of spondyloarthritis

In addition to altered peptidome due to HLA-B*27-*ERAP1/2* interaction, there is evidence to suggest that, overall, AS and HLA-B*27 may be associated with defective gut immunity, enabling greater bacterial transgression across the gut mucosa. AS gut bacterial profiles are distinct from healthy controls [18, 19, 20•, 21], with evidence suggesting that these differences are driven by immunogenetic effects of associated genetic variants rather than being solely secondary to the disease itself (Fig. 2) [20•, 22••]. Peptide elution studies have shown an enrichment of bacterial peptides homologous to known HLA-B27-presented epitopes in the stools of patients with AS, suggesting a failure in clearance of these bacteria, and CD8 responses were observed to several peptides presented by APCs-B*27 + , but not B27-negative donors, consistent with these peptides driving adaptive immune activation in AS [20•]. Furthermore, reactive arthritis (ReA), triggered upon bacterial infection, proceeds to AS in ~ 10% of patients [23]. Paradoxically, the onset of many immune-mediated diseases is thought to involve an infectious stage, during which dampened immune responsiveness would be disadvantageous in clearing inflammatory stimuli. This is a feature of AS, for example, as shown by reports of deficient control of EBV infection [24, 25]. The underlying mechanisms involve known genetic associations, such as *TLR4* [9] and *MEFV* [12•] involved in innate immune responses to bacterial components influencing a ‘reverse interferon signature’ in monocyte, macrophages and DCs from AS [26], as well as from HLA-B27/hb_2_m Tg rat model of SpA [27]. The initial infectious stage is followed by hyperactivation and disrupted self-tolerance in the context of innate immune activation and expansion of self-reactive Th17 cells or microbial mimicry (extensively reviewed [28]). CD8 T cells are therefore maintained in a state of heightened activation, potentially through persistent exposure to bacterial adjuvant, and fail to undergo senescence, leading to less productive responses to foreign antigen [25, 29••]. This further influences immune priming and results in greater proinflammatory cytokine (TNF, IL-23) production, contributing to the clinical features of joint and gut inflammation commonly found in AS [20•, 26, 27, 30]. In turn, exposure to gut-derived bacterial components lead to reduced integrity of the gut vascular barrier [31, 32], bacterial invasion of the intestinal epithelium [30] and disturbed microbiome that increase carriage of peptide pools presentable by HLA-B*27[20•], creating a deleterious cycle. These factors in combination affect the epitope repertoire available for T cell engagement [33, 34] and, consequently, alters the generation of adaptive immune diversity.

The exact mechanism by which HLA-B*27 induces AS is not known, but the leading theory proposes the ‘arthritogenic peptide hypothesis’, where unique microbial peptide(s) presented by HLA-B*27, similar and cross-reactive to human ‘self’ peptide(s), prime CD8 T cells to targets endogenous antigens found at the site of disease. In other inflammatory and autoimmune conditions, such as rheumatoid arthritis or type-1 diabetes, Tcell/pHLA biology is critical for initiation and propagation of disease [35, 36]. Yet, this knowledge is missing in AS. Studying the TCR repertoire provides a snapshot of an individual’s response to prior antigen exposure and can inform on the dynamics of immune responses, with application to immune monitoring in inflammatory diseases such as multiple sclerosis [37], autoimmune diseases [38], viral infection [39] or cancer [40] as well as biomarkers of treatment response [41]. In a variety of immune mediated diseases including type 1 diabetes [42], vitiligo [43] and narcolepsy [44], identification of expanded TCR clonotypes has assisted in demonstrating antigenic drivers of disease. Herein, we review available methodologies used in immune repertoire studies, up-to-date developments on AS and future directions towards the identification of the antigenic trigger in AS and its potential impact in AS therapeutics.

## Method for TCR Repertoire Studies

### Overview of TCR Structure

T cells recognise pHLA complexes through their T cell receptor (TCR). TCRs are highly diverse heterodimeric proteins consisting of a disulphide-linked α- and β-chains (αβ TCR), in the majority of T cells, or γδ chains (γδ TCR), expressed by T cells abundant at mucosal sites. Each chain is formed of a constant and a variable domain, and each variable domain contains three hypervariable loops or complementarity-determining regions (CDRs), which determine pHLA specificity. The variable regions of TCR β and γ chains are encoded by a number of variable (V), diversity (D) and joining (J) genes, while TCRα and δ chains are encoded by V-J genes only. TCR diversity is generated by 3 processes: (1) somatic recombination of VDJ gene fragments, (2) non-templated/random nucleotides insertion/deletions at the junction sites between the gene segments [45] and (3) pairing of α-β or γ-δ chains to form a functional TCR [46]. CDRs 1 and 2 are entirely encoded in germline DNA segment, whereas the CDR3a and CDR3b loops are products of junctional diversity, consequently being the most variable. These mechanisms yield an immense variety of TCR repertoire [47], estimated at around 10^11^ different clonotypes, with only fractional overlap in the TCR repertoires of any two individuals [48].

Each naïve T cell expresses a unique receptor with a unique spectrum of pHLA affinities [49]. When a TCR engages a pHLA on the surface of APCs, it becomes activated, inducing T cell proliferation leading to clonal populations that share the same TCR. In instances of strong selective pressure to common pHLA ligands among individuals (e.g. same infection/disease), similar clonally expanded TCRs arise, termed ‘public clonotypes’[50]. Whilst the majority of TCRs are rare [48], sharing of expanded TCR clonotypes between multiple individuals with a disease thus strongly supports a common antigenic drive [46, 51]. Similarly, alterations of the repertoire of public clonotypes among the general population reflects an underlying immune alteration that can be measured by TCR repertoire profiling [52].

### TCR Repertoire Profiling

The vast diversity of the immune repertoire creates major challenges for its analysis, both in terms of laboratory approaches to its characterisation and in the analysis of that data. In the past decades, different laboratory techniques have been developed to profile the TCR repertoire, which are reviewed in depth elsewhere [53]. Initial low-resolution studies enabled investigation of the diversity of V gene usage, including by employing monoclonal antibodies against V regions and flow cytometry, or by measurement of differences in the length of CDR3 sequences by quantitative PCR amplification of the region and spectratyping techniques. The development of next-generation sequencing (NGS) methods, however, has now enabled comprehensive repertoire profiling. Here, we will provide a brief description of the two main NGS approaches: bulk sequencing of pooled immune populations and single cell approaches.

### Population-Based Bulk-NGS Sequencing

Bulk methods are used to study repertoire diversity in larger cohorts. One of the initial considerations in such studies is the choice of starting material, gDNA or RNA [54]. Whilst using gDNA benefits from higher stability and better quantification of clonotype frequencies (single template per cell) [55], it has proven to be less sensitive, does not consider allelic exclusion (overestimating diversity) and is susceptible to sequencing errors due to introns and residuals of VDJ rearrangements. The opposite applies to RNA-based methods, which also account for level of expression. Because of the uniqueness of TCRβ CDR3 (higher combinatorial potential compared to α chains [54]) and its key role in determining antigen specificity, this has been the main target of interest.

The three most common methods for library construction are multiplex PCR, target enrichment and 5'RACE cDNA synthesis and nested PCR. Multiplex PCR approaches have been the most commonly used and can be applied to either gDNA or RNA. They use a set of primers for the constant region and all known V alleles to amplify the CDR3 region. Such approaches are thus limited in the detection of novel V alleles and are susceptible to amplification biases [56], although this can be corrected for by adjusting primer concentrations and experimental conditions [57] or by molecular barcoding [58]. Target enrichment methods use custom-designed RNA baits to hybridize with the gDNA/cDNA target, followed by a further amplification step involving fewer PCR cycles than is typically used in multiplex PCR, thus reducing susceptibility to amplification bias [59]. The third method, 5′RACE [60], uses a primer against the constant region of the TCR mRNA transcript for cDNA synthesis of the complete 5′ end. The terminal transferase activity of the reverse transcriptase enzyme incorporates additional dCTP bases at the 3′ end of the cDNA, allowing for a template-switch with an oligoG, completing the second cDNA strand [61]. This is followed by a nested PCR amplification using a single pair of primers (3′-C region and 5′-common adaptor), thus minimising amplification bias related to primer differences. The method enriches for all TCR variants in the sample (known and novel), a significant advantage over other methods [62, 63, 64]. Since a specific TCR may differ from another by only a single nucleotide and every method is susceptible to errors, to distinguish between errors and low-frequency clonotypes, the introduction of unique molecular identifiers (UMIs) during cDNA synthesis allows application of correction algorithms and for absolute counts to be obtained [65, 66]. Once the library is prepared, sequencing depth requirements depend on the aim of the experiment. For a disease-oriented analysis looking for clonally expanded TCRs, a low-coverage screening may even be enough, whilst deep sequencing would be required for a more complete and complex repertoire and to identify rare clones [64].

Primary analysis involves the recovery of TCR sequences from raw data, annotation and clonotype clustering and quantification, as summarized in previous reviews [54, 67, 68]. In brief, reads are initially processed to eliminate those with a high error rate, remove primer sequences and build consensus sequences (contigs) from aligning paired-end reads and multiple reads from the same cDNA molecule with matching UMIs. For each contig, the germline V(D)J alleles most likely involved in gene rearrangement are inferred from a reference set and annotated. The frequency of each identified rearranged sequence (clonotype) is then identified. From that point, output data (generally in the form of table) is used for secondary analysis and visualization. Of note, immune repertoire sequences can be also extrapolated from untargeted transcriptome sequencing [69]. Whilst this approach may be limited by sequencing depth (revealing only a fraction of the TCR diversity) and is susceptible to errors from short read sequence assembly, given the availability of transcriptomic datasets, it can be a useful tool to screen for highly prevalent clonotypes.

Secondary repertoire analysis uses descriptive statistical indices of diversity and homology and visualization methods that can be approached with the numerous omics tools developed by the scientific community over recent years [70, 71, 72] (https://omictools.com/repseq-category). While no framework is ubiquitous, the AIRR Community of the Antibody Society has established some standards for data representation (https://docs.airr-community.org/en/stable). Mathematical indexes used to measure diversity and convergence of immune repertoires [71] derive from ecology, to quantify ecosystem biodiversity based on the information theory. Diversity relates to the level of uncertainty that a TCR sequence, sorted from a repertoire, would belong to a certain T cell clone, which depends on the number of unique TCR sequences (richness) and their relative abundance (evenness). The different parameters used include the Shannon, Inverse-Simpson and Gini indices [73].

Differences between immune repertoire profiles from different samples/datasets, such as biased VDJ gene usage [74], suggest association of TCR clonotypes with disease pathogenesis. By providing a fingerprint of adaptive immunity, immune repertoire analysis can capture alterations of the immune fitness. Further analysis options address the antigen specificity of the TCR receptors. By evaluating patterns in sequence [75], structure [76] and physicochemical properties [77], clustering algorithms link TCRs to antigen specificity or to clinical outcomes. However, the prediction of a T cell epitome remains one of the biggest challenges of cellular and computational immunology, which requires additional experimental input [47]. Such input will probably derive from single-cell analysis, providing the full TCRαβ pair required for pHLA interaction, and subsequent functional studies identifying the complementary epitome and range of affinities.

### Single-Cell Transcriptomics

Single-cell TCR profiling has emerged in recent years, linking αβ chains with phenotyping by transcriptional profiling. scRNAseq was originally developed to obtain paired TCRαβ sequences [78]. Whilst it still can lead to straightforward antigen identification [79], further development of the technique now allows for surface marker phenotyping using DNA-barcoded antibodies, epigenetic profiling and even screening of a small number of peptide using MHC tetramers, thus expanding the potential to draw biological in vivo function of the clones. Among the biggest limitations are the high cost, limited number of covered cells and requirement of fresh material for the isolation and sorting of live cells (not always available). The different methodologies are reviewed elsewhere [80], but it is worth highlighting the major improvement in scTCR-Seq with emulsion-based approaches, which use water-in-oil emulsion droplets that trap single cells with small volumes of reagents, including the barcodes required for multiplexing.

## Findings to Date in AS (Implications for Aetiopathogenesis)

Multiple studies have now investigated T cell repertoire variation in AS and ReA, with consistent evidence of expansion of T cell clonotypes. Prior to the development of NGS, Marker-Hermann and colleagues performed seminal research in this field, following from their demonstration of CTL in ReA patients capable of reacting to the bacteria involved in triggering the ReA, and also self-reactive CTL [81]. CDR3 spectratyping and sequencing were then used to study TCR usage in patients with ReA, in comparison with healthy controls and rheumatoid arthritis patients [82]. Expansion of two TCRBV1-bearing T cells was demonstrated in synovial fluid of ReA but not healthy controls. TCRBV1 was defined by antibody staining (clone BL37.2) and is now known as TCRBV9 as defined by DNA sequence. In two patients, expansions involving two TCR clonotypes, bearing either the CDR3 sequence CAS-SVGLYSTDTQ or CAS-SPGLYSTDTQ, were identified [82]. A database search subsequently identified 148 HLA-B27/SpA-derived TCRB CDR3 sequences, from which a canonical TCRB CDR3 sequence, TCRBV1/23-CASSVG(V/I/L)(Y/F)STDTQYF-J2S3, was identified [83]. The database-derived HLA-B27/SpA sequences did not match those of CDR3 sequences from HLA-B27-responding clonotypes from healthy subjects, indicating a disease-association rather than simply being an HLA-B27-restricted motif. This TCR-CDR3 sequence matched 43/148 (29%) published ReA-derived CDR3 sequences from 12 different patients, but only 19/3799 (0.5%) other human TCRB sequences present in public sequence databases (odds ratio = 60, *P* < 10^−100^)[83].

The development of NGS enabled TCR profiling, as described above, encouraged further studies in this field. Using TCRB VDJ sequencing, Faham et al. studied 234 AS patient (192 HLA-B27 positive), and 227 controls (10 HLA-B27 positive), and confirmed increased carriage of a subset of six of the TCR clonotypes reported previously, and in total demonstrated 15 motifs enriched in the B27-positive AS patients, as compared to B27-positive healthy individuals (*P* = 0.001 and *P* = 0.049, respectively) [84]. The study involved only 10 HLA-B27 controls, and the findings for novel motifs that had not previously been reported were not analysed separately and thus require further validation. The previously reported SVGLYSTDTQ and TRBV9-SVGLYST-TRBVJ2-3 motifs were found in 30–39% of two sets of AS cases, and 0% of the overall control set (*P* = 4.6 × 10^−12^).

Using 5′-RACE, Komech et al. studied TCRB usage in blood and synovial fluid from 25 AS patients (24 HLA-B27 positive) and blood from 107 healthy controls (7 HLA-B27 positive, 8 HLA-B27 negative, 92 unknown HLA-B27 status). They also demonstrated expansion of TRBV9-CASSVGLYSTDTQYF-TRVBJ2-3. In total, eight TCR-CDR3 motifs were found to be expanded including three (CASSVGLFSTDTQYF, CASSVGLYSTDTQYF and CASSVGVYSTDTQYF) previously reported and confirmed by Faham et al. [84]. Seven of the eight expanded clonotypes were found amongst CD8-positive lymphocytes, consistent with their interaction with HLA-I alleles. Each of the eight AS-associated clonotypes were found in synovial fluid from four HLA-B*27-positive AS patients but not in synovial fluid from an HLA-B*27-negative patient.

Subsequent to this, Hanson et al. reported findings using bulk TCR repertoire profiling of sorted CD8 + and CD4 + PBMCs from 47 AS patients (37 HLA-B27-positive) and 38 healthy controls (20 HLA-B27-positive) [85••]. This study demonstrated expansion of both CD8 + and CD4 + TCR clonotypes in AS patients compared with HLA-B27-matched healthy controls. Ten closely related CD8 TCR clones were shown to be strongly associated with AS, found in all of 37 HLA-B27-positive AS patients, but only 4/19 HLA-B27-positive controls (*P* = 2.6 × 10^−6^). The previously reported CASSVGLFSTDTQYF motif was found in CD8 lymphocytes from 12 cases and no controls (P = 1.6 × 10^−5^).

Lastly, Zheng et al. used multiplex PCR and RNA-seq to profile TCR usage in peripheral blood and synovial joint fluid from axial spondyloarthritis patients (with either AS or non-radiographic disease) and healthy controls of unreported HLA-B27 status [86••]. This study suggested that expanded TCR clonotypes were seen amongst both CD4- and CD8-positive T cells, with identical expanded TCRB sequences in both CD4 and CD8 T cells observed. Comparison with previous findings confirmed expansion of the CAS***STDTQYF CDR3 motif in synovial joint fluid CD8 and CD4/8 T cells but not in CD4 + T cells. Additionally, expansion was observed with two other CDR3 motifs (CAS***SPLHF in SJF CD4/8 T cells, and CAS***GANVLTF in all CD4, CD8 and CD4/8 T cell populations from SJF). Whether these expansions are disease or HLA-B27 specific is not yet clear, but if they are truly disease-associated, then this is a particularly exciting confirmation, occurring in a completely different ancestral group to those previously studied, and likely involving patients with different HLA-B27 subtypes.

Overall, these findings provide strong evidence of expansion of a restricted set of CDR3 sequence–defined clonotypes particularly amongst CD8 lymphocytes in AS cases. This provides strong evidence supporting the arthritogenic-peptide model of how HLA-B27 induces AS.

## Immune Repertoire — Epitome Linking Approaches and Future Directions

**Fig. 3:**
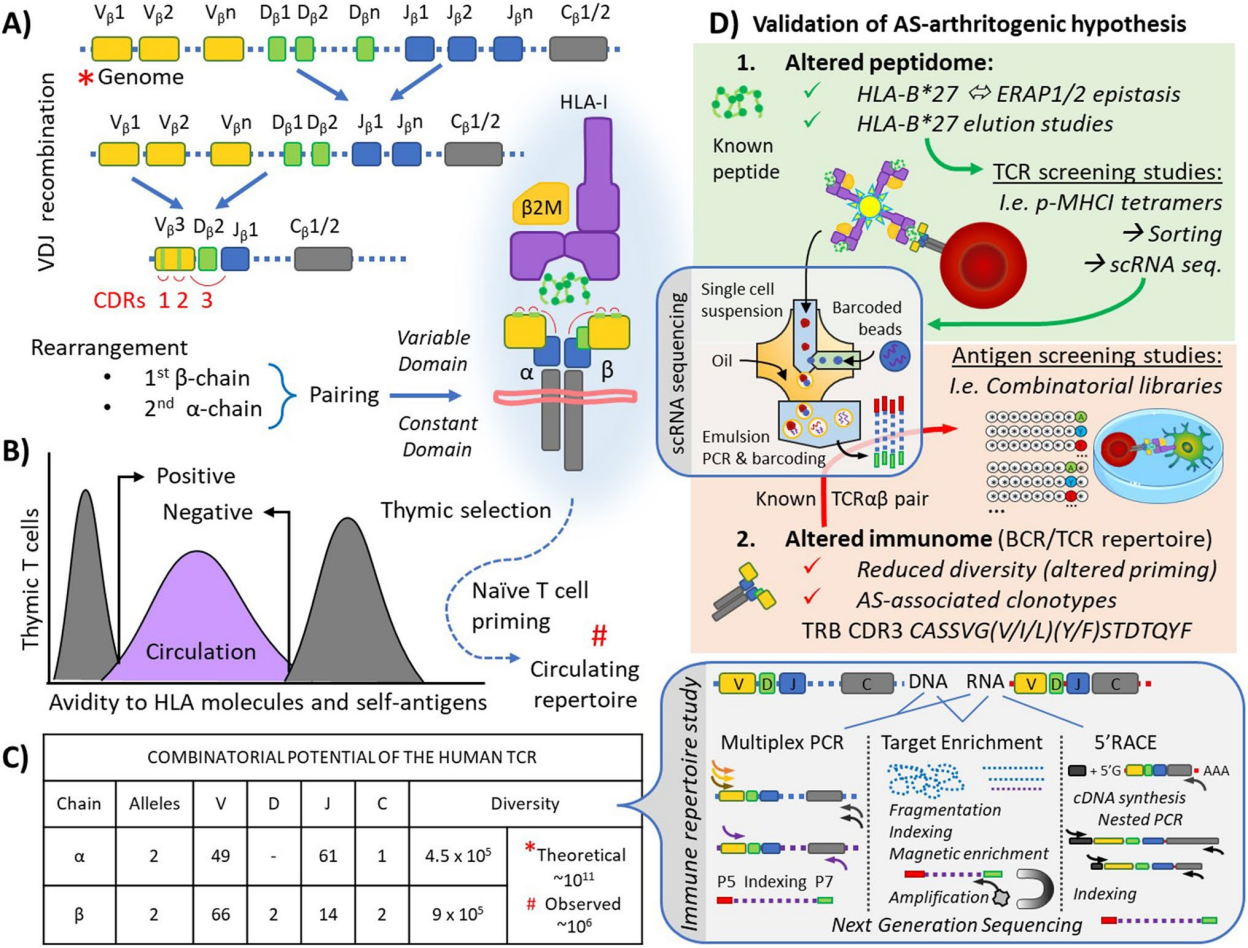
Immune repertoire diversity and validation of a TCR-pHLA association. **A** TCR diversity is generated by genetic recombination at the TCR locus. Within the thymus, rearrangement of V (variability), D (determining), J (joining) and C (constant) genes of the TCRB is followed by TCRA locus recombination. **B** Thymic selection of the recombined TCRαβ pair is based on their relative affinity for HLA molecules: a lower threshold to recognise an HLA allele (positive selection) and an upper threshold to avoid self-reactivity and autoimmunity against self-antigens (negative selection). The naïve T cell repertoire generated is further selected upon peripheral antigen encounters in the circulation and tissues, resulting in clonal proliferation and generation of adaptive memory. **C** The combinatorial potential of the TCR repertoire is required to cover the antigenic peptidome. However, the observed diversity is considerably lower than the theoretical diversity due to constraints such as possible and productive recombination, α-β pairing and HLA selection, in addition to sampling issues (much of the repertoire is represented by unique TCR sequences). **D** Immune repertoire studies address this diversity thanks to advances in next-generation sequencing (NGS) technologies. Either using DNA or RNA as starting material, TCR sequences can be enriched and prepared for sequencing using different methods, such as multiplex PCR amplification, target enrichment or 5′RACE (5′-rapid amplification of cDNA ends) and nested PCR amplification (the latter only for RNA input). Single-cell RNA sequencing (scRNA seq.) can also be used to study the immune repertoire of T cells clones. Whilst it has reduced capacity to capture the diversity due to limited cell input (max 10.000 cells), it can provide the TCRαβ pair chain sequences involved in the TCR-pHLA interaction (and transcriptome of each cells), being more suitable for the next steps of the validation of a TCR-pHLA interaction. According to pre-existing information related to the condition, (1) altered peptidome observed with, i.e. elution studies, or (2) altered immunome observed with repertoire studies, follow-up studies will seek to find the cognate TCR (TCR screening studies) or peptide (Antigen screening studies), respectively

Having identified and characterised an expanded clonotype, identifying the peptide(s) to which it is responding is a key translational step. The approaches taken to achieve this involve either TCR or antigen screening studies (Fig. 3).

### TCR Screening

TCR screening studies have been historically performed by co-culturing of mixed lymphocyte populations with antigen-pulsed APCs. Upon recognition, T cell activation can be evaluated by changes in surface markers, proliferation, cytokine production or cytolytic activity [87]. Activated clones can then be characterized, including by TCR sequencing. Increased throughput can be achieved with novel methods using fluorescently labelled soluble MHC tetramers loaded with the antigen, identifying antigen-specific T cells among a heterogenous population using flow cytometry [88]. Using tetramers labelled with different fluorochromes, a few different antigens can be studied per experiment, including the direct assessment of their phenotype with antibody panels, and by sorting and sequencing. Different variations of this approach have been developed. The use of nanoparticles harbouring > 10^4^ tetramers per particle increases the method’s sensitivity [89]. CyTOF detection with metal tags instead of fluorescence improves the phenotypic characterization, but at the cost of TCR definition by sequencing, as the cell is destroyed during detection [90]. DNA-tagged tetramers increase the number of epitopes that can be simultaneously evaluated [91] in combination with single-cell sequencing. Microfluidic devices have been developed enabling higher throughputs with reduced cell input requirements [89, 92]. These methods could be useful for the identification of high-avidity interactions, such as those of some neoantigens or microbe-specific T cells [93]. However, it is worth mentioning that the TCR-pHLA binding is of relatively low affinity and degenerate [48], so that many TCRs could recognize the same antigen, and many antigens are recognized by the same TCR. As such, more precise methods are required to investigate low-affinity repertoires, as are methods to overcome the difficulty of production, and lack of stability, of the multimers. Nevertheless, these technics are still in their infancy, so they hold promise as further developments overcome some of these limitations, such as their reliance in MHC tetramer technology.

### Antigen Screening

Antigen screening is a more suitable approach to follow-up for immune repertoire analysis that identifies clonal expansions associated to a particular condition, as in most cases, the antigen involved is unknown. This approach requires large numbers of cells expressing the TCRs of interest, which are typically obtained either from a natural source by immortalization or, more typically, by cloning into a cell line. When the origin of the epitope is suspected, the TCRs can be tested in vitro against a library of peptides or whole genome libraries of the suspected organism displayed on the surface of baculoviruses, yeast or mammalian cells [93, 94, 95, 96]. In a similar fashion to pHLA tetramers, soluble fluorescently tagged TCR tetramers can be used to screen these libraries, with the specific antigen being confirmed by sorting and sequencing of the host-display system [97]. However, this method entails the same challenges of generating the tetramers and the avidity of the TCR/pHLA physical interaction [47]. Cell-based strategies that evaluate triggered signals, rather than binding itself, can overcome these issues. Some recent developments use chimeric receptors that couple extracellular pHLA and intracellular TCR signalling domains in reporter systems, such as the SABRs (‘signalling and antigen-presenting bifunctional receptors’)[98] or pHLA/eTCRs[99]. However, the challenges of adapting those systems to the particular requirements of the study, such as specific HLA alleles (risk or protective), unconventional peptides or various TCRs variants, may limit their applicability and widespread adoption.

The most direct experimental method for antigen screening is based on the use of combinatorial peptide libraries (CPLs) [100]. These are highly complex mixtures of peptides systematically arranged so that each pool keeps one position defined whilst the others vary (termed as “positional scanning library”) [101]. Whilst complex, time consuming and costly, these studies allow the unbiased elucidation of T cell ligands and the functional characterization at each position, defining peptide variants that also induce activity [102]. Their use led to the discovery of TCR degeneracy [103] and to the identification of multiple ligands of different affinities for both MHC class I [104] and class II molecules [105]. CPLs have the advantages that they are more independent of the experimental system, applicable to different APC lines harbouring different sets of HLA molecules and can be used with a wide variety of readouts.

The sensitivity of the readout system may be a particularly relevant factor for a successful experimental detection of a TCR epitome, given than many TCR-pHLA interactions are of low affinity. Many studies use classical readouts, such as clonal expansion, surface marker upregulation or cytokines/granzyme B release [49], whist others have exploited “extravagant/unusual” outcomes of TCR-pHLA interaction, such as the transfer of membranous materials between T cells and APC (‘trogocytosis’) [106]. The employment of transcriptional reporter systems conveniently allows for unmanipulated straightforward quantification [107], an attractive option for large-scale studies.

## Repertoire Studies and the Development of AS Therapeutics

Repertoire analyses and the identification of TCR-pHLA associations involved in AS could not only help resolve the pathogenesis of AS, but would also be informative about multiple other HLA-I immune-mediated inflammatory diseases (IMIDs) such as psoriasis (HLA-Cw6), Behcet’s disease (HLA-B51) and birdshot retinopathy (HLA-A29). It is very likely that in these diseases, which are each strongly associated with different HLA Class I antigens and with ERAP1, the mechanism by which these associations operate to cause disease is similar. Identifying expanded TCR clonotypes and the antigen(s) driving these expansions have obvious potential therapeutic significance. Most directly, if these clonotypes are involved in driving inflammation in AS, then deleting them may be therapeutic. Phase 1 trials of an anti-TRBV9 monoclonal antibody, BCD-180, are underway in Russia on the bases of clonal expansions frequently carrying TRBV9 [108] (https://biocad.ru/pipelines/). Whilst this individual TRBV gene may be expanded in AS, there is substantial risk of targeting a large range of non-pathogenic TRBV9 TCRs, as well as missing AS-associated clones with a similar CDR3 but from a range of TRBV families. It is also not yet clear whether the expanded TRBV9-bearing cells are causing AS-associated inflammation or are involved in some unsuccessful disease-protective response. Thus, such approaches could lead to broader immunological effects and treatment-associated toxicities.

A more precise characterization of CD8 T cell clones that are uniquely expanded in HLA-B27 + AS patients could help to identify cellular biomarkers for improved diagnostics. Most importantly, this new knowledge would be critical for the development of targeted therapies informed by underlying disease mechanisms. The use of AS-expanded T cells to identify the antigenic driver(s) and its source, potentially cross-reactive with a bacterial antigen as suggested by the model of ReA and gut microbiome studies discussed above, raises the possibility of specific antimicrobial therapies to manage or prevent the disease. In rheumatoid arthritis, knowledge of immunopathogenic peptides, which are present 15 years before the onset of clinical symptoms, has revolutionised diagnosis [109] and opened the door for development of peptide-specific therapies [110]. In AS, microbiome studies [20•] and peptide elution studies from HLA-B27 [111, 112] could help to narrow down the number of peptides that require testing. Whilst an efficient approach, this could hinder the discovery of novel peptides of potential application to modulate the response of these pathogenic clones. For example, the antigen-specific response of AS-associated clones could be therapeutically inactivated by targeting the antigen to steady-state dendritic cells, which in turn regulate Ag-specific memory and effector T cell populations [113, 114]. Or as shown in ovalbumin-induced arthritis in mice [115], by TCR-gene transfer, the target of primary regulatory T cells could be redirected and adoptive therapy used to induce antigen-specific suppression of the pathology. These strategies would be specific to the condition, thus eluding off-target toxicity. In conclusion, the strategies informed by repertoire studies enable the prospect of preventative/early treatment for AS with potential for prolonged disease remissions [116].
